# How are the different specialties represented in the major journals in general medicine?

**DOI:** 10.1186/1472-6947-11-3

**Published:** 2011-01-21

**Authors:** Jean-Francois Gehanno, Joel Ladner, Laetitia Rollin, Badisse Dahamna, Stefan J Darmoni

**Affiliations:** 1 Institute of Occupational Medicine, Rouen University Hospital, 1 rue de Germont, Rouen, France; 2 Department of Epidemiology and Public Health, Rouen University Hospital, 1 rue de Germont, Rouen, France; 3 CISMeF team, LITIS Laboratory EA 4108, Rouen University Hospital, 1, rue de Germont, Rouen, France

## Abstract

**Background:**

General practitioners and medical specialists mainly rely on one "general medical" journal to keep their medical knowledge up to date. Nevertheless, it is not known if these journals display the same overview of the medical knowledge in different specialties. The aims of this study were to measure the relative weight of the different specialties in the major journals of general medicine, to evaluate the trends in these weights over a ten-year period and to compare the journals.

**Methods:**

The 14,091 articles published in The Lancet, the NEJM, the JAMA and the BMJ in 1997, 2002 and 2007 were analyzed. The relative weight of the medical specialities was determined by categorization of all the articles, using a categorization algorithm which inferred the medical specialties relevant to each article MEDLINE file from the MeSH terms used by the indexers of the US National Library of Medicine to describe each article.

**Results:**

The 14,091 articles included in our study were indexed by 22,155 major MeSH terms, which were categorized into 81 different medical specialties. Cardiology and Neurology were in the first 3 specialties in the 4 journals. Five and 15 specialties were systematically ranked in the first 10 and first 20 in the four journals respectively. Among the first 30 specialties, 23 were common to the four journals. For each speciality, the trends over a 10-year period were different from one journal to another, with no consistency and no obvious explanatory factor.

**Conclusions:**

Overall, the representation of many specialties in the four journals in general and internal medicine included in this study may differ, probably due to different editorial policies. Reading only one of these journals may provide a reliable but only partial overview.

## Background

In 1901, the Journal of the American Medical Association stated that "it is practically out of the question to be in touch with all the literature issued in any one department of medicine, because of the expense and time it would involve to attempt to master the stuff sufficiently well to separate the wheat from the chaff" [1]. This phenomenon has not waned since then and physicians face a dramatic challenge when they try to keep up to date with medical knowledge. General practitioners read or consult a small number of key journals on a regular basis with regard to their particular clinical practice, including usually one "major general medical journal". Specialists associate journals from their specialty and one or two "general journals". For example, British psychiatrists regularly read an average of 3 journals, among which at least one general medical journal. For example, 89% regularly read the British Medical Journal (BMJ) and 22% the Lancet [2]. A subsequent study among British Surgeons found percentages of 77.9% and 30.8% for the BMJ and the Lancet respectively, whereas the Journal of the American Medical Association (JAMA) or the New England Journal of Medicine (NEJM) were read by less than 1% of respondents [3]. Among American surgeons, besides specialized journals, 67% and 66% read the NEJM and the JAMA, respectively [4].

Since the clinicians rely mostly on one or two general medical journals to keep their medical knowledge up to date, it is worthwhile asking whether or not all medical journals provide a similar overview of the evolution of medical knowledge in each specialty.

We considered in this study the major journals, in terms of Impact Factor, of the subject category "Medicine, General & Internal" of the Journal of Citation Report.

The aim of this study was to measure the relative weight of the different specialties in these journals, *i.e. *the number of articles published concerning a specialty compared to the total number of articles published in one journal in one year. We also aimed at assessing the trends in these relative weights over a ten-year period and to compare the journals.

## Materials and methods

### Construction of the database

We selected four major periodicals, in terms of Impact Factor, in the subject category "Medicine, General & Internal" of the Journal of Citation Report, *i.e. *The Lancet, the New England Journal of Medicine (NEJM), the Journal of the American Medical Association (JAMA) and the British Medical Journal (BMJ). In order to have two US and 2 European journals, we included the 5^th ^journal of this category (BMJ) instead of the 4^th ^(Ann Intern Med).

The first step consisted of identifying all the articles published by these journals in 1997, 2002 and 2007 and indexed in the MEDLINE database (US National Library of Medicine, Bethesda, MD). We searched PubMed (URL: http://www.pubmed.org) with the journal title and each of these publication years, combined with the Boolean operator OR.

All the major keywords used by MEDLINE indexers to describe the articles retrieved at the end of this first stage were gathered to build a database, which was used to categorize the journals, one by one and one year at a time. This was done using a MEDLINE categorization algorithm that we had previously developed [5].

### Categorization of the articles

Categorization is designed to enhance resource description by organizing content description so as to enable the reader to quickly and easily grasp what a resource is about, and what are the main topics discussed in it.

In practice, this categorization algorithm lists the medical specialties relevant to a MEDLINE file by a decreasing order of their importance. These medical specialties are inferred from the MeSH (Medical Subject Headings) thesaurus from the US National Library of Medicine (NLM), and enhanced by the concept of metaterms (or "super-concepts") we previously developed [6]. Briefly, a metaterm is a medical specialty or a biological science (*e.g. *"cardiology" or "bacteriology"). For each existing metaterm (N = 104), one semantic link was created with at least one MeSH descriptor or qualifier. For example, the metaterm "psychiatry" is linked to the MeSH descriptors "psychiatry" and "psychiatric hospital" that belongs to a completely different tree structure within the MeSH. The list of metaterms and their respective semantic links with MeSH descriptors and qualifiers are available at the following URL: http://doccismef.chu-rouen.fr/liste_des_meta_termes_anglais.html.

The categorization algorithm uses all the semantic links existing between MeSH descriptors of an article indexed in the MEDLINE bibliographic database and metaterms to induce the list of metaterms for that particular article. This automatic categorization is based on the manual indexing of resources with MeSH (descriptors/qualifiers) pairs by NLM indexers. This process is performed recursively to obtain the list of metaterms related to any MEDLINE file obtained from any MEDLINE query.

We used only the major MeSH descriptors used by NLM indexers for the categorization.

If a MeSH descriptor has a link to several metaterms, it can induce more than one metaterm. For example, the descriptor thumb induces the metaterm "anatomy", and the descriptor "alcoholism" induces both the metaterms "psychiatry" and "toxicology".

Assume there are n major MeSH terms T1, T2, ... Tn, the categorization algorithm enables us to deduce k metaterms M1, M2, ... Mn from these sets of terms.

The categorization algorithm was applied to all the articles of our database. We then computed the number of occurrences of each metaterm for each journal and each of the years studied. We used only the metaterms related to medical or surgical specialties, and we have excluded the metaterms related to methods (*e.g. *"statistics") or laboratory tests.

## Results

Overall, 14,091 articles were published by the BMJ, the JAMA, the Lancet and the NEJM in 1997, 2002 and 2007. They were indexed by 141,474 MeSH terms, among which 22,155 were major MeSH terms. These major MeSH terms were linked by the categorization algorithm to 81 different metaterms representing medical or surgical specialties, each of them occurring between 2 ("thermal medicine") and 4101 ("cardiology") times. Overall, the major MeSH terms induced 62557 metaterms.

Table 1 shows the mean ranking, in terms of frequency, of the first thirty metaterms, and their frequency for each of the journals, on average for the three years studied. In the BMJ, JAMA and Lancet, "cardiology", "neurology" and "environment and public health" were in the first four metaterms, with "environment and public health" in the first rank in the BMJ and the JAMA, and in third rank in the Lancet. In the NEJM, this ranking was slightly different: the first three metaterms were respectively, "cardiology", "cancerology" and "neurology".

**Table 1:** Distribution in mean percentages and ranking of the first thirty occurrences of metaterms in the BMJ, JAMA, Lancet and NEJM for the years 1997, 2002 and 2007 cumulated

Specialties	BMJ		JAMA		LANCET		NEJM		Total	
	%	R	%	R	%	R	%	R	%	R
Cardiology	4.17%	2	6.49%	2	6.50%	2	9.15%	1	6.56%	1
Neurology	5.90%	3	5.66%	3	6.92%	1	6.14%	3	6.24%	2
Environment and public health	6.68%	1	6.53%	1	6.48%	3	2.81%	12	5.66%	3
Cancerology	2.70%	8	3.38%	8	5.17%	6	7.85%	2	4.85%	4
Infectious diseases	3.20%	5	4.05%	5	5.46%	4	5.31%	5	4.59%	5
Epidemiology	5.62%	4	5.13%	4	5.33%	5	1.92%	16	4.54%	6
Allergy and immunology	2.42%	14	2.32%	14	4.97%	7	5.61%	4	3.97%	7
Vascular medicine and surgery	2.04%	9	2.97%	9	3.50%	8	5.07%	6	3.42%	8
Hematology	1.73%	13	2.43%	13	3.28%	9	4.46%	7	3.01%	9
Surgery	2.53%	10	2.82%	10	2.39%	14	3.55%	10	2.79%	10
Gastroenterology	1.83%	22	1.81%	22	3.19%	10	3.89%	9	2.75%	11
Psychiatry	4.03%	6	3.88%	6	2.16%	17	1.30%	24	2.75%	12
Pulmonary disease	1.87%	15	2.22%	15	2.48%	13	4.03%	8	2.65%	13
Genetics	2.03%	17	2.04%	17	2.72%	12	2.95%	11	2.47%	14
Pediatrics	2.38%	18	2.00%	18	2.83%	11	2.18%	14	2.40%	15
Information science	3.68%	7	3.43%	7	1.90%	19	0.79%	32	2.37%	16
Economics	3.20%	11	2.77%	11	1.73%	22	1.22%	26	2.17%	17
Gynecology	2.43%	20	1.96%	20	2.24%	15	1.85%	17	2.14%	18
Obstetrics	2.45%	19	2.00%	19	2.20%	16	1.82%	18	2.13%	19
Endocrinology	1.35%	30	1.40%	30	1.93%	18	2.64%	13	1.85%	20
Rheumatology	1.50%	26	1.59%	26	1.73%	21	2.15%	15	1.75%	21
Toxicology	1.73%	21	1.81%	21	1.88%	20	0.89%	30	1.59%	22
Risk management	1.95%	16	2.16%	16	0.99%	31	1.30%	23	1.53%	23
Nutrition	1.40%	25	1.65%	25	1.66%	23	1.33%	22	1.52%	24
Law	3.00%	27	1.49%	27	0.95%	32	0.51%	40	1.45%	25
Ethics	1.80%	28	1.47%	28	1.43%	25	1.10%	28	1.45%	26
Forensic medicine	2.96%	29	1.41%	29	0.94%	33	0.39%	44	1.39%	27
Education	2.15%	12	2.51%	12	0.60%	37	0.71%	33	1.38%	28
Urology	0.98%	33	1.05%	33	1.25%	26	1.80%	19	1.28%	29
Addiction	1.90%	23	1.76%	23	1.04%	30	0.55%	38	1.27%	30

R: rank

Five metaterms were systematically ranked in the first 10 metaterms in the four journals and 15 metaterms were systematically ranked in the first 20 metaterms in the four journals. Among the first 30 metaterms, 23 were common to the four journals. Nevertheless, 4 metaterms were found in only one journal: "reproductive medicine" in the Lancet (1.51%, rank 24), "dermatology" in the NEJM (1.22%, rank 27), "nephrology" in the NEJM (1.24%, rank 25) and "thoracic and cardiovascular surgery" in the NEJM (1.61%, rank 20). The metaterm "education" was found in the first 30 metaterms in only two journals, the BMJ and the JAMA.

Tables 2 and 3 shows the evolution between 1997, 2002 and 2007 of the first 15 metaterms for each journal. The trend between 1997 and 2007 were either positive or negative, sometimes in very high proportions. For example, "vascular Medicine" and "cardiology" increased in the JAMA by respectively 231% and 161% and "genetic" decreased by 76% in the Lancet.

**Table 2:** Evolution of the relative weight of first 15 metaterms between 1997, 2002 and 2007 for the BMJ and the JAMA

	BMJ				JAMA			
Specialties	1997	2002	2007	Trend	1997	2002	2007	Trend
Cardiology	3.98%	4.28%	4.32%	+8%	4.28%	6.15%	11.2%	+161%
Neurology	7.06%	5.07%	5.17%	-27%	5.07%	7.54%	4.24%	-16%
Environment and public health	6.61%	6.54%	7.00%	+6%	6.54%	7.10%	5.70%	-13%
Cancerology	2.35%	2.72%	3.26%	+39%	2.72%	3.33%	4.73%	+74%
Infectious diseases	3.25%	2.97%	3.49%	+7%	2.97%	6.28%	3.13%	+5%
Epidemiology	5.38%	5.83%	5.69%	+6%	5.83%	3.80%	5.49%	-6%
Allergy and immunology	2.89%	1.96%	2.34%	-19%	1.96%	2.87%	2.26%	+16%
Vascular medicine and surgery	2.66%	1.62%	1.63%	-39%	1.62%	3.15%	5.35%	+231%
Haematology	1.47%	2.08%	1.60%	+9%	2.08%	1.96%	3.72%	+79%
Surgery	2.19%	2.76%	2.77%	+27%	2.76%	2.89%	2.82%	+2%
Gastroenterology	2.14%	1.78%	1.37%	-36%	1.78%	1.86%	1.77%	0%
Psychiatry	3.49%	4.12%	4.80%	+38%	4.12%	4.36%	2.71%	-34%
Pulmonary disease	1.91%	1.85%	1.83%	-4%	1.85%	2.58%	2.43%	+32%
Genetics	1.75%	1.92%	2.72%	+56%	1.92%	1.39%	3.13%	+63%
Pediatrics	2.09%	2.49%	2.72%	+30%	2.49%	1.96%	1.08%	-57%

Trend: increase or decrease between the relative weight of a metaterm (number of occurrences of the metaterm divided by the total number of metaterms for the journal and the year) between 1997 and 2007.

**Table 3:** Evolution of the relative weight of first 15 metaterms between 1997, 2002 and 2007 for the Lancet and the NEJM

	Lancet				NEJM			
Specialties	1997	2002	2007	Trend	1997	2002	2007	Trend
Cardiology	7.08%	6.85%	4.87%	-31%	9.86%	8.41%	9.23%	-6%
Neurology	7.94%	6.26%	6.25%	-21%	6.38%	6.21%	5.86%	-8%
Environment and public health	4.58%	6.64%	9.47%	+107%	2.33%	2.67%	3.35%	+44%
Cancerology	5.07%	5.61%	4.58%	-10%	7.61%	5.60%	10.18%	+34%
Infectious diseases	6.74%	4.98%	4.01%	-41%	5.46%	6.19%	4.34%	-20%
Epidemiology	4.21%	4.59%	8.50%	+102%	1.47%	2.38%	1.85%	+26%
Allergy and immunology	5.46%	5.41%	3.37%	-38%	5.81%	5.46%	5.56%	-4%
Vascular medicine and surgery	4.18%	3.45%	2.40%	-43%	5.48%	4.34%	5.41%	-1%
Haematology	3.19%	3.41%	3.19%	0%	4.64%	4.24%	4.51%	-3%
Surgery	2.17%	2.86%	1.96%	-10%	3.37%	3.97%	3.29%	-2%
Gastroenterology	3.50%	3.61%	1.94%	-45%	4.33%	3.97%	3.44%	-21%
Psychiatry	2.10%	1.86%	2.77%	+32%	1.01%	1.67%	1.18%	+17%
Pulmonary disease	2.43%	2.81%	2.03%	-17%	2.86%	4.54%	4.53%	+58%
Genetics	3.61%	2.90%	0.88%	-76%	2.77%	2.85%	3.18%	+15%
Pediatrics	2.62%	2.45%	3.81%	+45%	1.83%	2.83%	1.85%	+1%

Trend: increase or decrease between the relative weight of a metaterm (number of occurrences of the metaterm divided by the total number of metaterms for the journal and the year) between 1997 and 2007.

There was no homogeneous trend among journals, except for "neurology" and "gastroenterology", which decreased in the 4 journals. The analysis of the year 2002 showed that, most of the time, the evolution between 1997 and 2007 was not linear. For example, in the Lancet, "pediatrics" decreased between 1997 and 2002, but increased between 2002 and 2007.

## Discussion

### There are some differences in the topics covered by the major journals in general medicine

The main finding of this study was that the four major journals covered different topics.

When considering the main specialties concerned by the articles published in the four journals, we observed that five of them were constantly highly ranked, i.e. in the first ten: "cardiology", "neurology", "cancerology", "Infectious diseases" and "vascular medicine and surgery". Therefore, although these specialties were ranked slightly differently among the journals we studied, we can consider that they are equally represented. This was not the case for most of the other 76 specialties, which were very differently represented among the four journals. For example, "psychiatry" and "information sciences" were respectively the 6^th ^and 7^th ^specialties in the BMJ, and the 24^th ^and 32^nd ^in the NEJM. Therefore, although most readers assume that the main general medical journals provide a similar view of up to date medical knowledge that may be relevant for their practice, this assumption is not evidence based for many specialties.

### The trends are different

The trends over a ten-year period were different from one journal to another. Only "neurology" and "gastroenterology" displayed a consistent pattern, with a constant decrease in its relative weight in the four journals studied. For all the other specialties, their relative weight varied with non-coherent trends among journals, with some extreme situations such as for "cardiology" which increased by 161% between 1997 and 2007 in the JAMA but decreased by 31% in the Lancet in the same period. Therefore, one can question if the major journals of internal and general medicine display a similar picture in the evolution of medical knowledge.

Explanatory factors could include different editorial policies, official or not, or submission bias, since it is plausible that authors tend to submit their work to journals where articles dealing with similar topics are published.

### Limitations

We collected the data in the end of 2008. By using 2007 as the last year, we were certain not to have recent papers that could have been still "PubMed in process" citations. Such articles are not manually indexed with MeSH terms, subheadings and publication types, and therefore are not possible to map with metaterms. We therefore had a complete year of publication for each journal. Since the cited half-life of these journals is between 7.5 and 9.4 years, we chose 1997, i.e. 10 years before 2007 as the historical point. We then used 2002 as a mid term to see if the trends between 1997 and 2007 were linear. We did not included all the years between 1997 and 2007 because of the amount of data would have overloaded the categorization algorithm.

Our study relied on the use of the concept of metaterms. The validity of the semantic links between MEDLINE terms/subheadings and the metaterms may be questioned. Nevertheless, the semantic links where created based on the known how of professional librarians and medical experts, with the help of the Network of NLM using the Medlib-L listserv. Furthermore, this validity was recently compared to NLM Journal Descriptors to categorize scientific articles and no significant difference was observed [7].

Although there is no similar tool available, to our knowledge, Bodenreider described a similar categorization algorithm based on UMLS semantics and MeSH disease categories (N = 22) [8]. The UMLS algorithm performed better than the algorithm we used (relevance of 92% vs. precision and recall of 81% and 93%) [6]. However, the MEDLINE categorization algorithm we used was able to classify scientific articles among 115 different specialties whereas the Bodenreider's algorithm works with 22 MeSH disease categories. Furthermore, the metaterms are broader than the MeSH disease categories, each of them being included in at least one metaterm. Finally, the same algorithm was applied to the four journals studied and the comparisons between journals are therefore considered reliable.

The fact that our algorithm was restricted to the use of major MeSH terms allowed us to categorize articles only according to the main topics discussed in the articles.

### Why should these results be taken into consideration by readers?

The consequences of the fact that the relative weight of the different specialties may be different among the four most popular general medical journals should be taken into consideration, in an era of Evidence Based medicine.

Therefore, readers that rely on only one of these well known international journals should be aware that they may obtain a reliable, although partial, view on the evolution of medical knowledge in each specialty.

One could hope that some readers might be aware of this phenomenon and choose the general medical journal they read according to its specialization. Nevertheless, few studies have attempted to identify the reasons why a physician chooses a journal over another. Some physicians may take the impact factor into consideration, but it is in fact not related to the reading habits of US surgeons or British psychiatrists [2,4]. The journal's country of origin in fact seems to be the most important factor since physicians usually read journals from their own country [2,4]. The cost of the journals is probably also an explanatory factor but it is linked to the "country effect". For example, the members of the American Medical Association receive the JAMA, without subscription, whereas the members of the British Medical Association receive the BMJ free of charge.

## Conclusion

Overall, the representation of many specialties in the four journals in general and internal medicine included in this study may differ, probably due to different editorial policies. Since it is wishful thinking to suppose that physicians have enough time to keep in touch with the overwhelming amount of medical information, they should in fact be aware of these differences. Some initiatives, such as the "All you need to read in the other general journals" of the BMJ could help the practitioners to be reasonably informed, with a good cost-effective ratio.

## Competing interests

The authors declare that they have no competing interest.

## Authors' contributions

JFG designed the study. JFG, LR and SD collected the data. BD built the MEDLINE categorizer and entered the data in the categorizer. JL performed the statistical analysis. JFG, JL, LR and SD analysed the data. All authors read and approved the final manuscript.

## Pre-publication history

The pre-publication history for this paper can be accessed here:

http://www.biomedcentral.com/1472-6947/11/3/prepub
